# Root-Zone Nitrogen Fertilization Increases Oilseed Rape Yield: Reprogramming Rhizosphere N-Cycling and Strengthening Soil–Plant Coupling

**DOI:** 10.3390/plants15081137

**Published:** 2026-04-08

**Authors:** Liang Cheng, Quanjie Shen, Yifan Wang

**Affiliations:** 1 Key Laboratory for Improving Quality and Productivity of Arable Land of Yunnan Province, College of Resources and Environment, Yunnan Agricultural University, Kunming 650201, China; 2 State Key Laboratory of Soil and Sustainable Agriculture, Institute of Soil Science, Chinese Academy of Sciences, Nanjing 211135, China; 3 University of Chinese Academy of Sciences, Beijing 100049, China

**Keywords:** root-zone nitrogen fertilization, rhizosphere, soil nitrogen cycling, metagenomics, microbial functional genes, *Brassica napus* L.

## Abstract

Root-zone nitrogen fertilization (RZF) can increase crop N uptake and yield, yet the underlying rhizosphere N-cycling functional mechanisms remain insufficiently resolved. In a field experiment with winter oilseed rape (*Brassica napus* L.), RZF was compared with conventional fertilization (CF) under the same N input rates, alongside a zero-N control (N0). Compared with CF, RZF significantly increased seed yield (by 0.44 t ha^−1^) and aboveground N uptake (by 20.45 kg ha^−1^), while simultaneously enriching rhizosphere mineral N pools (NH_4_^+^–N and NO_3_^−^–N by 54.50% and 56.02%, respectively). Shotgun metagenomics revealed that RZF reprogrammed rhizosphere N-cycling functional potential, characterized by enhanced nitrogen fixation, reduced nitrification and denitrification, and a tendency toward increased assimilatory nitrate reduction. These module-level shifts were supported by concordant changes in key functional genes, indicating greater genetic potential for N retention and assimilation (*nifD*, *glnA*, *gltB*, *nasA*, *napB*, *nrfA*) and reduced potential for nitrification- and denitrification-driven N losses (*amoB*/*C*, *narI*, *nirK*, *norB*). Taxonomic composition analysis showed enrichment of *Bradyrhizobium* and suppression of key nitrifier taxa (*Nitrosospira* and a *Nitrososphaeraceae*-affiliated taxon) under RZF. Rhizosphere pH exhibited the strongest Mantel correlation with multiple N-cycling modules, and rhizosphere available N (AN; sum of NH_4_^+^–N and NO_3_^−^–N) was positively associated with plant N traits and yield. Structural equation modeling supported a pathway in which a functional balance index (retention/assimilation vs. loss/oxidation) increased AN (0.22), and AN strongly promoted yield (0.90). Collectively, these results elucidate a rhizosphere-centered mechanism whereby localized N placement strengthens soil–plant N coupling and enhances crop productivity through reprogramming microbial N-cycling functional potentials, positioning rhizosphere N processes as a key mechanistic bridge for microbiome-informed optimization of root-zone fertilization.

## 1. Introduction

Synthetic N fertilization is a cornerstone of global food and oilseed production. However, nitrogen use efficiency in intensive agriculture remains critically low—with only 30–50% of applied N typically recovered in harvested products [1,2]. This inefficiency results in substantial N surpluses and adverse environmental impacts, such as nitrate leaching and nitrous oxide emissions, particularly in intensive agroecosystems [3]. Winter oilseed rape (*Brassica napus* L.), a major global oil crop, requires high N inputs to achieve optimal yields. Thus, enhancing crop N uptake and synchronizing N supply with plant demand are essential to reconcile high productivity with environmental sustainability [4,5]. Conventional N management, which often relies on broadcast or shallow-banded applications, frequently creates a spatial mismatch between soil N supply and root uptake [6]. This mismatch leads to transient pools of mineral N in surface soils that are highly prone to loss [7,8], highlighting the need for N management strategies that simultaneously increase crop N uptake and minimize N losses.

Root-zone nitrogen fertilization (RZF), involving deep and concentrated application of N fertilizers into the active rooting zone, has emerged as a promising spatial management strategy [6,7]. By creating localized high-concentration zones, RZF reduces N exposure to surface runoff and positions nutrients closer to actively growing roots [6,9,10]. The agronomic benefits of RZF—including enhanced plant N uptake, increased yield, and optimized soil mineral N dynamics—have been demonstrated across various crop systems, alongside reductions in gaseous N losses [9,10,11,12], with growing evidence supporting its efficacy in oilseed rape [13].

The rhizosphere serves as a critical microbial and biogeochemical hotspot, where root activity drives the transformation and ultimate fate of soil nitrogen [14]. Within this dynamic interface, microbial communities mediate the full nitrogen cycle [15]. The functional balance between two broad categories of microbial pathways—loss-driven processes (e.g., nitrification and denitrification) and retention- or assimilation-driven processes (e.g., nitrogen fixation and assimilatory nitrate reduction)—is largely regulated by the abundance and expression of specific functional genes [16,17]. This balance determines the fate of applied nitrogen—whether it is retained for crop uptake or lost to the environment—thereby governing its partitioning between plant-accessible pools and loss pathways [18,19].

From an agronomic management perspective, by physically concentrating fertilizers, RZF creates an intense, spatially heterogeneous nutrient hotspot within the dynamic root zone. This precise spatial placement generates steep chemical concentration gradients that govern the remodeling of local microbial communities. Recent studies have demonstrated that localized N fertilization strategies, such as band placement or deep hole application, can profoundly impact specific functional microbial guilds (e.g., ammonia oxidizers) and drive distinct N-cycling processes compared to conventional non-localized fertilization [20,21,22,23]. However, most of these investigations have focused primarily on bulk soil environments and relied on targeted analyses of specific microbial taxa. Consequently, from a system-level metagenomic perspective, it remains poorly understood how the agronomic practice of spatial N fertilizer placement reshapes the overall microbial N-cycling functional potential within the rhizosphere—the critical plant–microbe interface. Specifically, it is unclear whether the concentrated N zones created by RZF shift comprehensive rhizosphere microbial N-cycling networks toward retention/assimilation pathways (which sustain plant-available N pools) or toward loss/oxidation pathways (which accelerate N loss). Unraveling this functional balance is essential, as it ultimately determines the coupling efficiency between rhizosphere N availability and plant performance.

To address this gap, conventional N application (CF) and root-zone N fertilization (RZF) were compared in a field winter oilseed rape system using a combination of plant N uptake and yield assessments, rhizosphere soil property measurements, and rhizosphere metagenomic analyses. The present objectives were to: (i) evaluate the effects of N fertilization on the rhizosphere soil mineral N (NH_4_^+^, NO_3_^−^), plant N uptake, and yield; (ii) characterize the response of rhizosphere N-cycling gene repertoires, functional pathways, and microbial networks to different fertilization strategies; and (iii) elucidate the linkages between rhizosphere soil mineral N pools, rhizosphere N-cycling functional potential, and plant performance. It was hypothesized that root-zone fertilization acts as an ecological filter, selecting for a rhizosphere microbiome enriched in nitrogen retention/assimilation-associated functional potential rather than loss/oxidation-associated potential, thereby increasing plant-accessible rhizosphere mineral N and strengthening soil–plant N coupling.

## 2. Results

### 2.1. RZF Increases Seed Yield and Plant N Uptake

**Figure 1:**
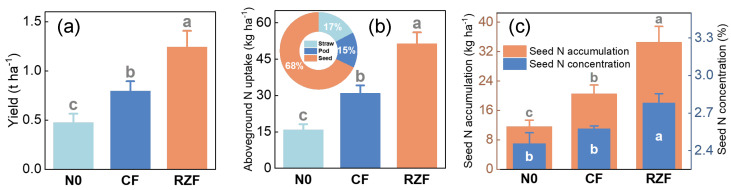
Effects of N application methods on yield and plant N traits of winter oilseed rape. (**a**) Seed yield. (**b**) Aboveground N uptake. (**c**) Seed N concentration and seed N accumulation. N0, zero-N control; CF, conventional fertilization; RZF, root-zone N fertilization. Values are means ± SE (n = 4). Different letters indicate significant differences among treatments according to LSD test (*p* < 0.05).

Root-zone N fertilization (RZF) significantly increased seed yield, aboveground N uptake, and seed N status compared with both the zero-N control (N0) and conventional fertilization (CF) (Figure 1). Specifically, seed yield under RZF was higher than that under N0 and CF by 0.76 and 0.44 t ha^−1^, respectively. Aboveground N uptake under RZF increased by 35.49 kg ha^−1^ compared with N0 and by 20.45 kg ha^−1^ compared with CF. Nitrogen was mainly allocated to seeds, which accounted for 68% of total aboveground N. Seed N concentration under RZF was 13.23% and 8.03% higher than that under N0 and CF, respectively, while seed N accumulation increased by 22.96 kg ha^−1^ compared with N0 and 14.06 kg ha^−1^ compared with CF (Figure 1).

### 2.2. RZF Reshapes Rhizosphere Properties and Elevates Available N Linked to Yield

**Figure 2:**
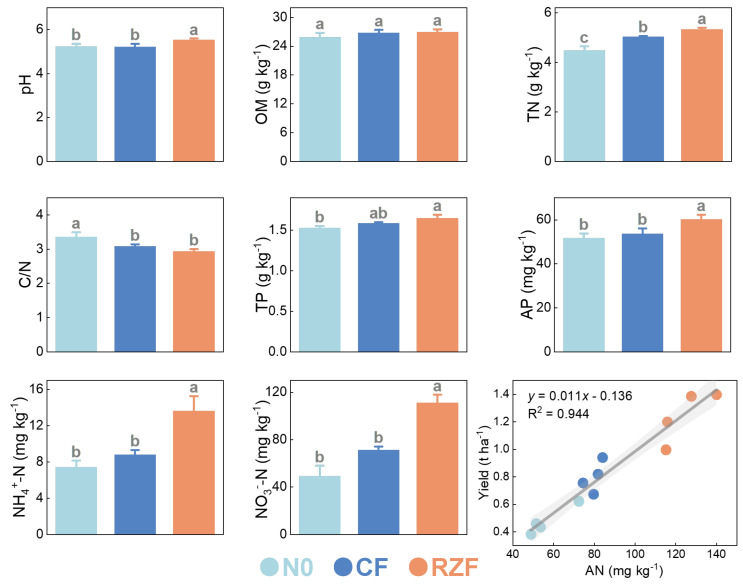
Rhizosphere soil properties and the relationship between available N and yield. OM, organic matter; TN, total N; TP, total P; AP, available P; AN, available N (AN = NH_4_^+^–N + NO_3_^−^–N). N0, zero-N control; CF, conventional fertilization; RZF, root-zone N fertilization. Values are means ± SD (n = 4). Different letters indicate significant differences among treatments according to LSD test (*p* < 0.05).

Root-zone N fertilization significantly altered rhizosphere soil physicochemical properties and mineral N pools (Figure 2). Rhizosphere pH under RZF increased by 0.29 and 0.30 units compared with N0 and CF, respectively. Organic matter (OM) showed no significant differences among treatments. Total N (TN) under RZF increased by 19.2% compared with N0 and by 5.85% compared with CF. The C/N ratio under RZF was 13.63% lower than that under N0 but did not differ from CF. Total P (TP) under RZF increased by 8.03% compared with N0 but did not statistically differ from CF, whereas available P (AP) under RZF increased by 16.35% and 12.19% compared with N0 and CF, respectively. Mineral N pools were also elevated under RZF: NH_4_^+^–N increased by 82.96% compared with N0 and 54.50% compared with CF, and NO_3_^−^–N increased by 125.83% compared with N0 and 56.02% compared with CF. Notably, rhizosphere available N (AN = NH_4_^+^–N + NO_3_^−^–N) exhibited a strong positive linear relationship with seed yield (R^2^ = 0.944, *p* < 0.01) (Figure 2).

### 2.3. N-Cycling KO Diversity and Composition Differ Across N Application Methods

**Figure 3:**
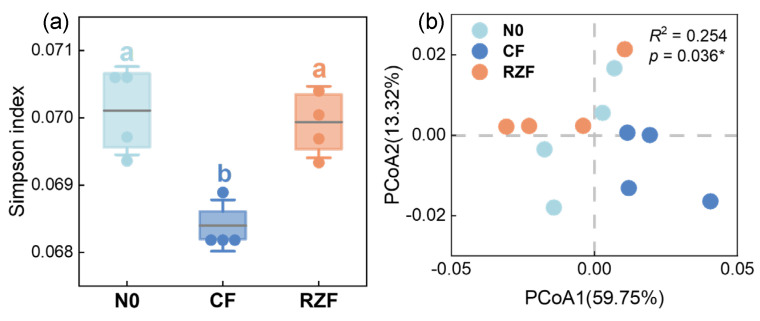
Alpha diversity and beta diversity of rhizosphere N-cycling KOs. (**a**) Simpson index of N-cycling KOs. (**b**) PCoA based on Bray–Curtis distances, with treatment effects tested by PERMANOVA. Alpha diversity was assessed using the original Simpson index (*D*), where higher values represent lower overall functional diversity. N0, zero-N control; CF, conventional fertilization; RZF, root-zone N fertilization. Values are means ± SD (n = 4). Different letters indicate significant differences among treatments according to Dunn’s post hoc test (*p* < 0.05). *: *p* < 0.05.

RZF significantly increased the Simpson index of N-cycling KOs compared with CF, while no significant difference was detected between RZF and N0 (Figure 3a), indicating reduced KO diversity under RZF compared with CF. Principal coordinates analysis (PCoA) based on Bray–Curtis dissimilarities showed a clear separation in N-cycling KO composition among treatments. PERMANOVA confirmed that N application method significantly affected the overall N-cycling KO profile (*p* < 0.05), with a larger separation between RZF and CF in the ordination (Figure 3b).

### 2.4. RZF Reconfigures Rhizosphere N-Cycling Functional Modules and Key Genes

**Figure 4:**
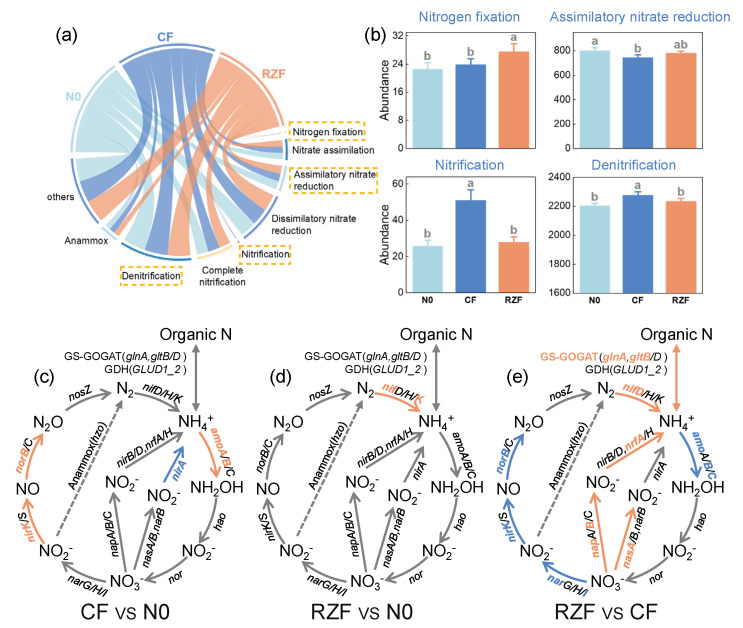
Reconfiguring of rhizosphere N-cycling functional modules and genes under different N application methods. (**a**) Chord diagram showing the relative abundance of rhizosphere N-cycling functional modules; yellow dashed boxes denote modules differing among treatments. (**b**) Module abundance (RPKM) of nitrogen fixation, assimilatory nitrate reduction, nitrification, and denitrification. (**c**–**e**) Differential abundance of N-cycling genes (RPKM) for CF vs. N0 (**c**), RZF vs. N0 (**d**), and RZF vs. CF (**e**); orange indicates significantly upregulated genes, blue indicates significantly downregulated genes, and grey indicates no significant difference. N0, zero-N control; CF, conventional fertilization; RZF, root-zone N fertilization. Values are means ± SD (n = 4). Different letters indicate significant differences among treatments according to Dunn’s post hoc test (*p* < 0.05).

At the functional module level, RZF significantly reconfigured rhizosphere N metabolism potential (Figure 4a,b). The nitrogen fixation module (RPKM) under RZF increased by 22.55% and 15.67% compared with N0 and CF, respectively. Assimilatory nitrate reduction showed no significant difference between treatments but exhibited an upward trend under RZF compared with CF (increase of 4.68%). In contrast, nitrification did not differ between RZF and N0 but decreased significantly under RZF compared with CF (−45.32%). Denitrification showed no difference between RZF and N0 and decreased under RZF compared with CF although the magnitude was small (−4.83%). Differential gene analyses further demonstrated treatment-dependent shifts in key N-cycling functional genes (Figure 4c–e). Compared with N0, CF significantly increased *amoA*/*amoB*, *nirK*, and *norB* while reducing *nirA*. Compared with N0, RZF significantly increased *nifK* only. Importantly, compared with CF, RZF significantly increased genes associated with ammonium assimilation (*glnA*, *gltB*), nitrogen fixation (*nifD*), and nitrate/nitrite reduction linked to N retention (*nasA*, *napB*, *nrfA*), while significantly decreasing nitrification (*amoB*/*amoC*) and denitrification genes (*narI*, *nirK*, *norB*). Together, these results indicate that RZF promotes N-input and N-retention–oriented function potentials while suppressing oxidative and loss-prone function potentials in the rhizosphere.

### 2.5. Taxonomic Features in N-Cycling Microbiomes and Co-Occurrence Networks

**Figure 5:**
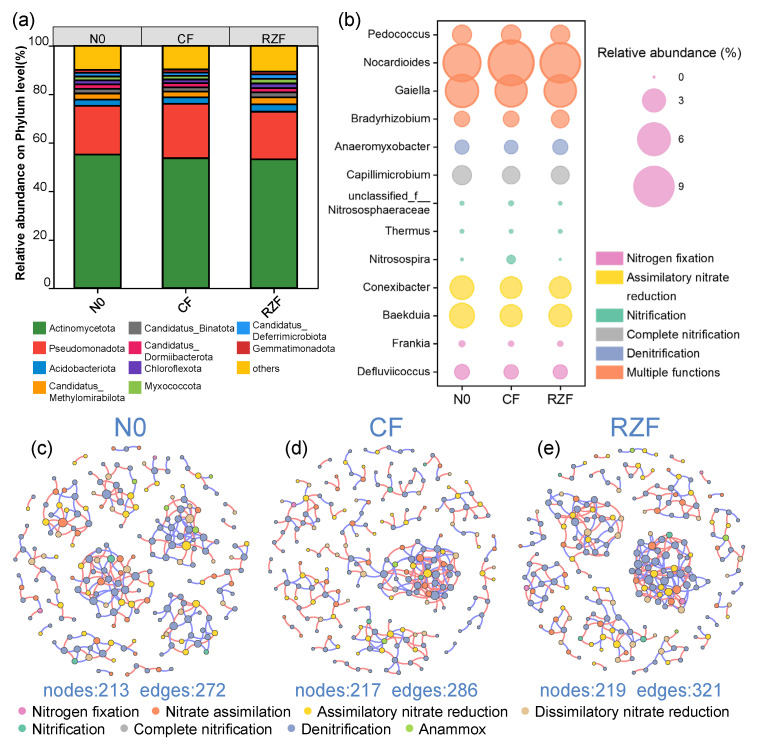
Taxonomic composition of N-cycling microbiomes and co-occurrence networks. (**a**) Phylum-level composition of N-cycling microbiomes in the rhizosphere. (**b**) Relative abundance of the top five genera contributing to each N-cycling module. (**c**–**e**) Species-level co-occurrence networks of N-cycling taxa for N0 (**c**), CF (**d**), and RZF (**e**). N0, zero-N control; CF, conventional fertilization; RZF, root-zone N fertilization.

Across treatments, the ten most abundant phyla within the N-cycling microbiomes—*Actinomycetota*, *Proteobacteria*, *Acidobacteriota*, *Candidatus Methylomirabilota*, *Candidatus Binatota*, *Candidatus Dormiibacterota*, *Chloroflexota*, *Myxococcota*, *Candidatus Deferrimicrobiota*, and *Gemmatimonadota*—collectively accounted for approximately 90% of the total relative abundance (Figure 5a). Several phyla differed significantly between CF and RZF, including *Proteobacteria*, *Nitrospirota*, *Nitrososphaerota*, *Armatimonadota*, and *Candidatus Walliibacteriota* (Figure S1). To link taxa to functions, the top five genera contributing to each module were determined (Figure 5b and Table S2). Under RZF, *Bradyrhizobium*—highly contributing to nitrogen fixation and nitrate assimilation—showed a significantly higher relative abundance than under CF. In contrast, *Nitrosospira* and an unclassified genus within *Nitrososphaeraceae*—key contributors to nitrification—were significantly reduced under RZF compared with CF (Table S2). At the species level, co-occurrence networks constructed using taxa annotated with N-cycling KOs showed higher edge number, average degree, density, and clustering coefficient under RZF than under N0 and CF, along with lower average path length and diameter (Figure 5c–e and Table S3).

### 2.6. Environmental Drivers and Soil–Plant Coupling Revealed by Mantel Tests, Correlation Analysis, and SEM

**Figure 6:**
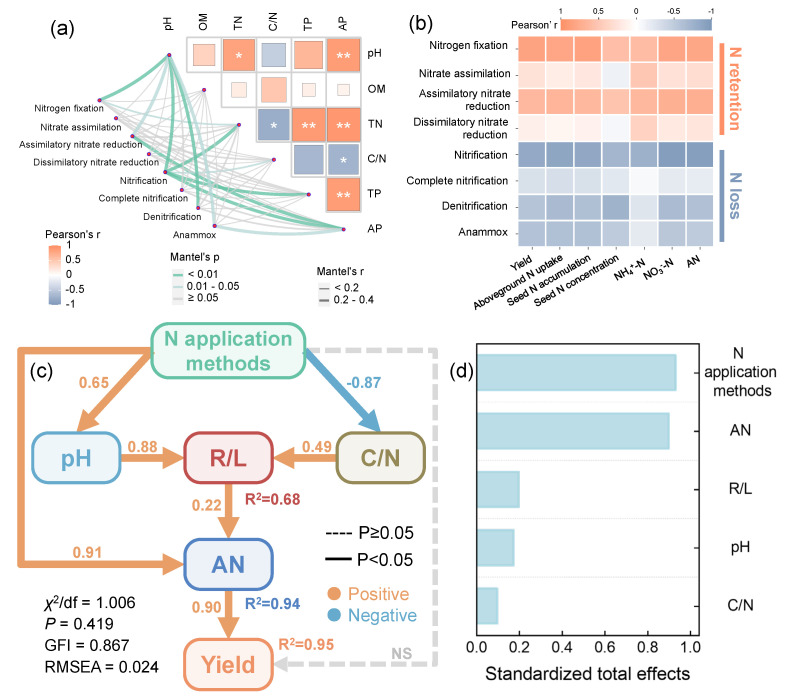
Mantel tests, correlation analysis, and structural equation model (SEM) link rhizosphere properties to N-cycling modules, mineral N availability, and yield. (**a**) Mantel tests between rhizosphere environmental variables and N-cycling modules. (**b**) Correlations between N-cycling modules and rhizosphere mineral N pools (NH_4_^+^–N, NO_3_^−^–N, AN), plant N traits (aboveground N uptake, seed N accumulation, seed N concentration), and yield. (**c**) SEM depicting the structural relationships among N application methods, rhizosphere properties, functional balance (R/L), rhizosphere available N (AN), and yield; numbers on arrows are standardized path coefficients. (**d**) Standardized total effects of each factor on yield. OM, organic matter; TN, total N; TP, total P; AP, available P; AN, available N (NH_4_^+^–N + NO_3_^−^–N); R/L, ratio of N-retention to N-loss module abundance (RPKM-based). *: *p* < 0.05, **: *p* < 0.01.

Mantel tests revealed significant associations between rhizosphere environmental variables and the composition of N-cycling modules (Figure 6a and Table S4). Rhizosphere pH was significantly correlated with nitrogen fixation, assimilatory nitrate reduction, nitrification, denitrification, and anammox modules (*p* < 0.05), whereas OM showed no significant relationships. TN and C/N were significantly correlated with nitrification. TP was significantly associated with nitrification, while AP was significantly associated with assimilatory nitrate reduction, nitrification, and anammox. Correlation analysis further indicated that yield, aboveground N uptake, seed N accumulation, and seed N concentration, as well as rhizosphere NH_4_^+^–N, NO_3_^−^–N, and AN, were positively associated with “N-retention” modules (nitrogen fixation, nitrate assimilation, assimilatory nitrate reduction, and dissimilatory nitrate reduction) and negatively associated with “N-loss” modules (nitrification, complete nitrification, denitrification, and anammox) (Figure 6b and Table S5). Structural equation model (SEM) integrated these relationships into an overall framework (Figure 6c,d). N application methods exerted direct effects on rhizosphere pH (0.65) and C/N (−0.87), which in turn regulated the ratio of N-retention to N-loss functional potential (R/L). The R/L ratio had a significant direct effect on AN (0.22), while N application methods also directly increased AN (0.91). Ultimately, rhizosphere AN had a strong positive effect on yield (0.90), whereas N application methods did not directly affect yield in the model (*p* > 0.05). Total effect analysis showed that N application methods had the largest positive total effect on yield (0.93), followed by AN (0.90), R/L (0.20), pH (0.17), and C/N (0.10) (Figure 6d and Table S6).

## 3. Discussion

### 3.1. Root-Zone N Fertilization Reprograms Rhizosphere N-Cycling Functional Potential

Root-zone N fertilization (RZF) induced pronounced shifts in the overall profile of rhizosphere N-cycling functional genes. Principal coordinates analysis (PCoA) based on KEGG N-cycling KOs clearly separated CF and RZF along the first two axes, and PERMANOVA confirmed significant treatment effects on the N-cycling functional gene profile (Figure 3b). At the module level, RZF significantly increased the abundance of the nitrogen fixation module and significantly decreased the nitrification and denitrification modules compared with CF, while the assimilatory nitrate reduction module exhibited a non-significant trend towards higher abundance under RZF (Figure 4a,b). Furthermore, the significantly higher Simpson index (*D*) under RZF (Figure 3a) indicates reduced overall functional diversity but a highly specialized microbial community. Specifically, the functional potential shifted overwhelmingly toward N fixation and assimilation rather than nitrification and denitrification, driving a functional reorientation toward mineral N retention over loss within the soil–plant system [24].

This module-level reprogramming was further supported by consistent gene-level shifts. RZF increased the relative abundances of key genes in the GS–GOGAT ammonium assimilation pathway (*glnA*, *gltB*), nitrogen fixation (*nifD*), and nitrate/nitrite reduction pathways that regenerate NH_4_^+^ (*nasA*, *napB*, *nrfA*), while reducing marker genes for nitrification (*amoB*, *amoC*) and denitrification (*narI*, *nirK*, *norB*) relative to CF (Figure 4e). Together, the coordinated upregulation of N fixation and assimilation/retention-associated genes alongside the downregulation of nitrification and denitrification genes demonstrates that RZF promotes a rhizosphere metagenomic functional profile with enhanced potential to incorporate mineral N into microbial biomass and organic N pools, while limiting genetic potentials that drive NH_4_^+^ oxidation and the formation of terminal NO_3_^−^ or other loss-prone end products [25,26].

Mechanistically, these shifts can be interpreted as a consequence of localized N placement, which redistributes mineral N and enhances microscale heterogeneity within the root zone. In localized root-zone fertilization systems, subsurface point placement of N fertilizer concentrates mineral N into microsites within the active rooting layer, creating stronger spatial gradients than shallow, surface-applied, or diffuse placement [7]. Studies on deep or side-deep placement have shown that such subsurface N concentration localizes mineral N closer to active roots, promotes localized root growth, and increases plant N uptake compared with more diffuse placement [8,11,13]. Furthermore, localized N concentration generates steep microscale gradients in mineral N availability and associated soil chemistry, which can impose selective pressures on microbial communities and, in some systems, has been linked to suppressed nitrifier abundance or activity within fertilizer-affected microsites [20,21,22,23].

As roots grow into and around fertilizer-influenced soil volumes, fertilizer-affected microsites increasingly overlap with zones of intense rhizodeposition, forming rhizosphere domains enriched simultaneously in mineral N and root-derived labile C. Within the “hotspot/hot-moment” framework, these domains can be viewed as rhizosphere hotspots where elevated resource supply and intense microbial–root activity co-occur [27]. Under such conditions, strong environmental filtering is expected to favor taxa and module-level functional potential capable of rapidly exploiting coupled C and N availability, thereby reducing the ecological advantage of ammonia oxidizers and other oxidation/loss pathways [28]. This filtering mechanism elucidates the observed shifts in alpha diversity (Figure 3a). Conventional broadcast fertilization (CF) homogenizes the root zone, activating diverse N-loss pathways and maintaining higher overall functional diversity. In contrast, RZF spatially constrains these generalized reactions. By selectively enriching N retention and assimilation pathways, RZF reduces the evenness of the N-cycling gene pool. This targeted dominance is precisely reflected by the higher Simpson index (*D*) observed under RZF. Overall, this framework is consistent with the observed increase in nitrogen retention-prone potentials and the concurrent reduction in nitrogen loss-prone potentials under RZF.

Quantitative support for this environmental-filtering interpretation was provided by Mantel tests linking rhizosphere properties to N-cycling module dissimilarities (Figure 6a and Table S4). Among measured variables, rhizosphere pH showed the strongest and most significant association with the N-cycling modules, while total N (TN) and the C/N ratio also exhibited significant correlations. This pattern suggests that the metagenome-level functional shift was structured not only by localized mineral N availability, but also along gradients of rhizosphere pH and N availability. Soil pH is widely recognized as a key regulator of nitrifier and denitrifier communities and their functional outcomes, influencing substrate speciation, enzyme activity, and niche differentiation of these guilds [29,30]. Likewise, soil N status and C/N balance can influence microbial allocation between N immobilization and mineralization, thereby reshaping N-cycling functional potential by shifting the balance between C and N limitation [31,32,33]. Taken together, these results support the view that RZF acts as an environmental filter in the rhizosphere, primarily by modifying the pH–N resource context and thus favoring pathways that retain N in reduced or assimilated forms over pathways that promote NH_4_^+^ oxidation and the formation of loss-prone end products [15,18,34].

### 3.2. Taxonomic Shifts and Network Patterns of the Rhizosphere N-Cycling Microbiome Under RZF

The metagenome-inferred shift in N-cycling functional potential under RZF was accompanied by changes in the dominant taxa harboring N-cycling genes, consistent with fertilization-associated restructuring of the rhizosphere microbiome under spatially concentrated N supply [19,35]. Across taxonomic levels, RZF was consistently associated with a lower relative abundance of ammonia-oxidizing taxa and a higher relative abundance of taxa linked to N assimilation and retention, providing a coherent taxonomic basis for the functional shifts reported in Section 3.1. At the phylum level, RZF decreased the relative abundance of *Proteobacteria* and *Nitrososphaerota* while increasing *Nitrospirota* in the rhizosphere (Figure 5a and Figure S2). At the genus level, shifts were concentrated in high-contribution genera aligned with specific N-cycling modules: *Bradyrhizobium* increased under RZF, contributing strongly to nitrogen fixation and nitrate assimilation, whereas nitrifier-associated genera (*Nitrosospira* and an unclassified genus within *Nitrososphaeraceae*) declined relative to CF (Figure 5b and Table S2).

Notably, the concurrent decline in *Nitrososphaerota* (ammonia-oxidizing archaea (AOA)-associated taxa) and bacterial nitrifier genera (ammonia-oxidizing bacteria (AOB)-associated taxa) corresponds with the reduced nitrification module and lower abundance of nitrification marker genes (e.g., *amo* genes) under RZF (Figure 4b,e), indicating that the decrease in nitrification potential was supported by coordinated reductions in both archaeal and bacterial nitrifier taxa rather than a single group alone. This pattern aligns with the established ecological sensitivity of AOA and AOB to N form, substrate concentration, and microhabitat conditions [20,36,37]. Conversely, the increase in *Bradyrhizobium* is consistent with its recognized role as a key diazotrophic lineage in plant-associated environments and its frequent linkage to N_2_-fixation potential and plant–microbe N interactions [38]. More broadly, fertilization and N enrichment have been reported to reshape soil microbial communities by suppressing subsets of nitrifier-associated taxa and favoring those adapted to higher resource availability across cropland soils [39,40,41]. Overall, these patterns suggest that the functional differences between RZF and CF were accompanied by a consistent shift in taxa most strongly associated with nitrification versus N assimilation/retention functions in the rhizosphere, consistent with previous soil N-cycling studies demonstrating taxon–function coherence when functional genes are integrated with community composition [26,42].

Network analysis was conducted as supporting evidence. Co-occurrence networks were constructed using species-level taxa annotated with N-cycling KOs (relative abundance > 0.01%; |r| > 0.7). Compared with CF, the RZF network exhibited a higher average degree and number of edges and shorter average path length (Figure 5c–e and Table S3), indicating a more connected association structure among N-cycling taxa. Similar increases in network connectivity and shorter path lengths under contrasting land use and N fertilization regimes have been reported and are commonly interpreted as reflecting tighter association structures among functionally relevant taxa in response to management-driven resource redistribution [25,40,43]. Thus, at the network level, these findings provide ecological evidence that localized N placement is accompanied by a reorganization of associations among N-cycling taxa in the rhizosphere. Because co-occurrence network inference can be sensitive to sample size and compositionality, these network patterns are interpreted as descriptive evidence of association restructuring rather than direct proof of stability or causality [44].

In summary, RZF was associated with coordinated taxonomic shifts consistent with the observed functional changes, including reduced representation of ammonia-oxidizing taxa and increased contributions from taxa associated with N retention-oriented functions. This taxon–function coherence provides an ecological framework consistent with the subsequent increases in rhizosphere mineral N pools and plant N uptake reported in later sections.

### 3.3. Rhizosphere N-Cycling Functions Mediate Soil–Plant N Coupling

Fertilizer placement altered rhizosphere mineral N pools, which were closely linked to plant yield. Compared with CF and N0, RZF increased rhizosphere TN as well as mineral N pools (NH_4_^+^–N, NO_3_^−^–N, and AN) (Figure 1 and Figure 2). Yield and plant N traits were strongly and positively correlated with rhizosphere soil mineral N (NH_4_^+^–N, NO_3_^−^–N, AN) (Figure 2 and Figure S1). This relationship is consistent with the well-established role of rhizosphere mineral N supply as an immediate constraint on plant N acquisition and yield formation in fertilized cropping systems [34,45]. To evaluate whether fertilization-induced shifts in metagenome-inferred N-cycling functional potential co-varied with rhizosphere AN and yield, associations among N-cycling modules, rhizosphere mineral N pools, and plant performance were examined.

Correlation analyses showed that N retention/assimilation modules (nitrogen fixation, nitrate assimilation, assimilatory nitrate reduction, and dissimilatory nitrate reduction) were positively correlated with rhizosphere NH_4_^+^–N, NO_3_^−^–N and AN, as well as plant N traits and yield, whereas N loss/oxidation modules (nitrification, complete nitrification, denitrification, and anammox) exhibited the opposite pattern (Figure 6b and Table S5). Similar co-variation between N-cycling functional gene profiles, mineral N pools, and crop N status has been reported in metagenomic studies of fertilized agricultural soils [25,42,46,47]. Importantly, these findings indicate that variation in rhizosphere mineral N pools and plant performance aligns with the coordinated reconfiguration of multiple N-cycling modules, rather than with any single module in isolation.

Because rhizosphere NH_4_^+^–N and NO_3_^−^–N represent net outcomes of simultaneous microbial transformations, root uptake, and physicochemical processes, microbial process rates cannot be inferred directly from mineral N pool data alone [32,45]. Taken together, the metagenomic patterns, rhizosphere mineral N pools, and structural equation model (SEM) pathways support an interpretation consistent with a rhizosphere N retention–turnover mechanism that may help maintain plant-accessible mineral N under localized fertilizer placement. Here, “retention” refers to microbial immobilization that temporarily stores mineral N in microbial biomass and organic N pools under conditions of high N availability, whereas “turnover” refers to the subsequent recycling and remineralization of this immobilized N, thereby replenishing plant-available NH_4_^+^-N and NO_3_^−^-N over time. This interpretation is consistent with evidence that microbial immobilization can transiently retain fertilizer-derived N, while microbial turnover and mineralization can subsequently return N to plant-available pools, particularly in rhizosphere environments characterized by high C inputs and elevated microbial activity [48,49]. Concurrently, the observed decrease in functional potential (e.g., for nitrification and denitrification) under RZF is consistent with a shift away from pathways that channel reactive N toward loss-prone transformations, thereby favoring the maintenance of plant-available mineral N within the rhizosphere [18].

Within this context, SEM provides complementary, model-based support by integrating N application methods, functional balance (R/L), rhizosphere AN, and yield into a unified structural framework (Figure 6c and Table S6). Specifically, R/L showed a significant direct effect on rhizosphere AN (standardized path coefficient = 0.22), and AN exerted a strong and highly significant direct effect on yield (0.90). Total effects further indicated positive contributions of N application methods (0.93), AN (0.90), and R/L (0.20) to yield (Figure 6d and Table S6). Together, the SEM supports a metagenome-inferred functional pathway in which N application methods regulate rhizosphere functional balance (R/L) and mineral N availability (AN). Among the rhizosphere-level variables included, AN emerged as the strongest mediator of yield, whereas N application methods acted as the upstream management driver with the largest total effect.

### 3.4. Implications for Rhizosphere N Cycling Under Root-Zone N Fertilization

Under the same N input, RZF was associated with higher seed yield and greater aboveground N uptake in winter oilseed rape (Figure 1 and Figure 2). Overall, the results support a rhizosphere-centered cascade in which RZF is linked to shifts in rhizosphere N-cycling functional potential, accompanying changes in plant-available rhizosphere mineral N pools, and strengthened soil–plant N coupling, collectively consistent with enhanced plant N accumulation (particularly in seeds) and higher yield. Rather than acting as sole and direct determinants, these N-cycling processes may represent an intermediate mechanistic link connecting fertilizer placement with plant N uptake and yield.

From a management perspective, these findings indicate that RZF reshapes rhizosphere N-cycling functional potential and associated transformations at the root–soil interface. The observed shift in functional potential toward retention/assimilation-associated modules and away from nitrification- and denitrification-associated modules suggests that localized N placement may help maintain plant-available mineral N in the rhizosphere through a shift in rhizosphere N-cycling functional potential. In this context, metagenome-derived functional indices—such as the balance between “retention/assimilation” and “loss/oxidation” functional potential—may serve as useful indicators for evaluating fertilization performance across sites, seasons, and management regimes. In addition, the associations between rhizosphere TP/AP and multiple N-cycling modules suggest that rhizosphere-scale N–P coupling may co-vary with N-cycling responses under localized N placement, consistent with evidence that P availability can constrain microbial N transformations through energetic and stoichiometric regulation [50,51,52].

However, several limitations warrant consideration. This study was conducted at a single site and during a single growing season, and the generalizability of the observed associations among N-cycling functional potential, mineral N pools, and yield across different soils and climatic conditions remains to be established. Metagenomic profiles reflect functional potential rather than actual process rates, and rhizosphere NH_4_^+^–N and NO_3_^−^–N pools represent net outcomes of microbial transformations, root uptake, and physicochemical dynamics. Moreover, key loss pathways (e.g., N_2_O emissions and leaching) and gross N transformation rates were not quantified, limiting direct inference from functional shifts to environmental outcomes. Nevertheless, the current metagenomic evidence provides a critical mechanistic foundation for understanding how localized fertilizer placement orchestrates rhizosphere microbial functions to support crop productivity.

## 4. Materials and Methods

### 4.1. Experimental Site and Experimental Design

The field experiment with winter oilseed rape (*Brassica napus* L.) was conducted from November 2024 to May 2025 in Gusheng Village, Dali City, Yunnan Province, Southwest China (25°48′51″ N, 100°08′36″ E). The site is subject to a subtropical plateau monsoon climate characterized by distinct dry and wet seasons, with a mean annual temperature of 15.1 °C and precipitation of 1379.1 mm. The soil type is paddy soil, with the following properties in the 0–20 cm profile: pH 6.51, organic matter (OM) 55.0 g kg^−1^, total nitrogen (TN) 4.45 g kg^−1^, available phosphorus (AP) 85.1 mg kg^−1^, and available potassium (AK) 139 mg kg^−1^.

**Figure 7:**
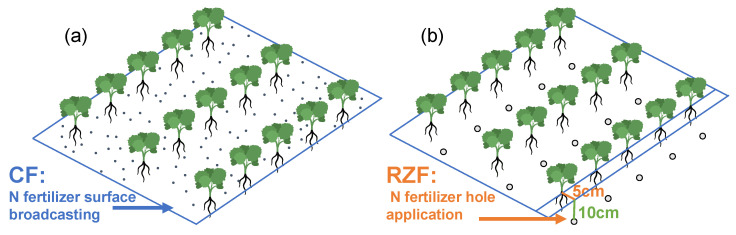
Schematic diagram of conventional N fertilization (CF) (**a**) and root-zone N fertilization (RZF) (**b**).

This study employed a randomized complete block design with three treatments and four replicates to compare localized root-zone fertilization (RZF) with conventional fertilization (CF). The treatments were: (1) Zero-nitrogen control (N0): no N application; (2) CF: N fertilizer split-applied (60% basal applied 3 days post-transplanting at the seedling establishment stage, and 40% topdressed at the post-winter bolting stage) by surface broadcasting; and (3) RZF: N fertilizer applied as a one-time basal application (applied simultaneously with the CF basal application). Specifically, to ensure precise spatial control within the relatively small experimental plots, the RZF operation was performed manually. The fertilizer was applied to every individual plant by deep point-placement into a specific hole located 5 cm laterally from the plant base at a depth of 10 cm (Figure 7). The total fertilizer application rates were strictly identical between the CF and RZF treatments: 225 kg N ha^−1^ (urea, 46% N), 75 kg P_2_O_5_ ha^−1^ (calcium superphosphate, 12% P_2_O_5_), and 90 kg K_2_O ha^−1^ (potassium chloride, 60% K_2_O). Phosphate and potassium fertilizers were applied as basal fertilizers and broadcast in all treatments (concurrently with the basal N application). The cultivar ‘Yunyouza 15’ was transplanted in early November after nursery cultivation in October. Consequently, all basal applications (the one-time RZF application and the 60% CF basal application) were completed before the overwintering period. Each plot was 12.6 m^2^ (7.0 m × 1.8 m) with a spacing of 0.20 m × 0.30 m. Field management practices, including irrigation and pest control, followed local agronomic standards.

### 4.2. Plant and Soil Sampling

Sampling was conducted at the crop maturity stage. Five representative plants were randomly selected from each plot. For rhizosphere soil collection, loosely adhering soil was removed by shaking, and the tightly adhering rhizosphere soil (<2 mm from the root surface) was collected using a sterile brush. The rhizosphere soil samples from the five plants were pooled into a sterile bag, homogenized, and transported to the laboratory on dry ice. Samples were sieved (2 mm mesh) to remove debris and divided into three parts: one stored at −80 °C for metagenomic DNA extraction; one stored at 4 °C for soil available nitrogen (NH_4_^+^-N and NO_3_^−^-N); and the final portion air-dried and sieved (0.149 mm) for physicochemical analysis. Concurrently, the plants were separated into roots, stems, pods, and seeds, and their fresh weights were immediately recorded. Plant tissues were subjected to 105 °C for 30 min to deactivate enzymes, followed by oven-drying at 70 °C to a constant weight. Finally, the dried samples were ground and passed through a 0.149 mm sieve for nitrogen analysis.

### 4.3. Yield and Plant N

At harvest, all remaining plants in each plot were harvested to determine the final seed yield. For nutrient analysis, the ground plant samples were digested using the sulfuric acid–hydrogen peroxide (H_2_SO_4_-H_2_O_2_) method. Nitrogen concentrations were quantified using a SmartChem 200 autoanalyzer (Westco Scientific Instruments, Brookfield, CT, USA). Plant N accumulation was calculated as the product of dry biomass and N concentration for each organ. Total aboveground N uptake was calculated as the sum of N accumulation in stems, pods, and seeds.

### 4.4. Soil Properties

Soil pH was measured in a 1:2.5 (*w*/*v*) soil-water suspension using a pH meter (Mettler Toledo, Greifensee, Switzerland). Soil NH_4_^+^-N and NO_3_^−^-N were extracted by shaking with 2 mol L^−1^ KCl for 1 h, and the filtrates were analyzed colorimetrically using a SmartChem 200 discrete autoanalyzer. Soil available phosphorus (AP) was extracted with 0.5 mol L^−1^ NaHCO_3_ (pH 8.5) and measured by the molybdenum-blue method using a UV-Vis spectrophotometer (Thermo Fisher Scientific, Waltham, MA, USA). Organic matter (OM) was determined using the K_2_Cr_2_O_7_ oxidation-titration method. For total nutrients, soil samples were digested with H_2_SO_4_-HClO_4_. Total N (TN) was measured using the autoanalyzer, and total P (TP) was determined by the molybdenum-blue method. All physicochemical analyses followed standard protocols [53].

### 4.5. Soil DNA Extraction and Metagenomic Sequencing

Genomic DNA was extracted from 0.2 g of rhizosphere soil using the E.Z.N.A.^®^ Soil DNA Kit (Omega Bio-tek, Norcross, GA, USA). DNA concentration and purity were quantified using a Synergy HTX multi-mode reader (BioTek, Winooski, VT, USA) and a NanoDrop 2000 spectrophotometer (Thermo Fisher Scientific, Waltham, MA, USA), while integrity was verified via 1% agarose gel electrophoresis. For library preparation, DNA was fragmented to ~400 bp using a Covaris M220 ultrasonic system (Gene Company, Hong Kong, China). Sequencing libraries were constructed with the NEXTFLEX™ Rapid DNA-Seq Kit (Bio Scientific, Avondale, AZ, USA) and sequenced on an Illumina NovaSeq™ X Plus platform (Majorbio, Shanghai, China) using the NovaSeq X Series 25B Reagent Kit.

### 4.6. Bioinformatic Analysis

Bioinformatic analysis was conducted on the Majorbio Cloud Platform [54]. The sequencing generated an average of 73.52 million raw reads per sample. Raw reads were quality-filtered by fastp (v0.20.0, https://github.com/OpenGene/fastp (accessed on 24 December 2025)) to remove adapters and low-quality bases (average quality value < 20), resulting in an average of 72.87 million high-quality reads per sample. High-quality reads were assembled into contigs using MEGAHIT (v1.1.2, https://github.com/voutcn/megahit (accessed on 24 December 2025)) [55] with a multiple k-mer strategy (--k-list 21,41,61,81,101,121), retaining contigs ≥ 500 bp. Open reading frames (ORFs) were predicted using Prodigal (v2.6.3, https://github.com/hyattpd/Prodigal (accessed on 24 December 2025)) [56] running in metagenome mode (-p meta), and ORFs ≥ 100 bp were selected. A non-redundant gene catalog was constructed via CD-HIT [57] (v4.6.1 http://weizhongli-lab.org/cd-hit/ (accessed on 24 December 2025)) using thresholds of 90% sequence identity and 90% alignment coverage (parameters: -c 0.9 -aS 0.9). Gene abundance was calculated as Reads Per Kilobase per Million mapped reads (RPKM) by mapping reads to the catalog using SOAPaligner (v2.21, https://github.com/ShujiaHuang/SOAPaligner (accessed on 24 December 2025)) [58] with an alignment threshold strictly set to ≥ 95% sequence identity to control for mismatches. Taxonomic and functional annotations were assigned by aligning genes against the NCBI NR and KEGG databases (version 20241007) using DIAMOND (v2.0.13, e-value < 10^−5^). Targeted nitrogen-cycling genes of functional modules were selected based on previous studies [25,42,59] and are listed in Table S1. In this study, “retention/assimilation” groups were defined as nitrogen fixation, nitrate assimilation, assimilatory nitrate reduction, and dissimilatory nitrate reduction, whereas “loss/oxidation” groups were defined as nitrification, complete nitrification, denitrification, and anammox. The functional balance index R/L was calculated as the ratio of the summed functional gene abundances (RPKM) of retention/assimilation modules to those of loss/oxidation modules. The relative contribution of a taxon to a specific function was calculated as its abundance divided by the total abundance of all taxa performing that function [60]. The raw metagenomic sequencing data have been deposited in the NCBI Sequence Read Archive (SRA) under the accession number PRJNA1428161.

### 4.7. Statistical Analysis

All statistical analyses were primarily performed using R software (v4.3.2) and AMOS 23.0 (IBM, Armonk, NY, USA). Data normality and homogeneity of variance were assessed using the Shapiro–Wilk and Levene’s tests, respectively, by the rstatix package (v0.7.3). For normally distributed and homoscedastic data, differences among treatments were evaluated using one-way ANOVA followed by the LSD post hoc test (*p* < 0.05). Conversely, for non-normal or heteroscedastic data, the Kruskal–Wallis test was employed, followed by Dunn’s post hoc test with False Discovery Rate (FDR) correction (*p* < 0.05). Alpha diversity of N-cycling KOs was assessed using the original Simpson index (*D*), with higher values indicating lower overall functional diversity. To evaluate differences in functional community structure, Bray–Curtis distance matrices were calculated based on N-cycling KO abundances (RPKM) and visualized using Principal Coordinates Analysis (PCoA). Significant differences were tested using Permutational Multivariate Analysis of Variance (PERMANOVA) with the adonis function in the vegan package (v2.6-4). Co-occurrence networks were constructed to explore interactions among N-cycling microbial species. This analysis was restricted to species with a relative abundance > 0.01% to reduce noise. Networks were built based on robust pairwise Spearman correlations (|r| > 0.7, *p* < 0.01). Topological indices were calculated using the igraph package (v2.1.4), and visualizations were generated using Chiplot (https://www.chiplot.online/ (accessed on 8 January 2026)). Furthermore, the relationships between soil environmental factors and N-cycling functional modules were assessed using Mantel tests in the linkET package (v0.0.7.4). Finally, structural equation modeling (SEM) was developed using AMOS to elucidate potential causal pathways among treatments, soil properties, N-cycling functional modules, and crop productivity.

## 5. Conclusions

Root-zone N fertilization (RZF) effectively enhanced seed yield and aboveground N uptake of winter oilseed rape under the same N input. Metagenomic analyses revealed that RZF reprogrammed rhizosphere N-cycling functional potential, characterized by enhanced nitrogen fixation potential, a trend toward higher assimilatory nitrate reduction, and reduced nitrification and denitrification potentials. This functional shift was driven by a taxonomic turnover that enriched N-assimilating bacteria while suppressing key nitrifiers. Structural equation modeling confirmed that the balance between N retention/assimilation and loss/oxidation functional potential significantly increased rhizosphere available N, which subsequently drove crop yield. Overall, the present results support a rhizosphere-centered pathway in which localized N placement reshapes microbial N-cycling potential and rhizosphere mineral N pools, thereby strengthening soil–plant N coupling and enhancing crop N uptake and productivity. These findings position the rhizosphere microbiome as a mechanistic bridge linking fertilizer placement to crop performance, providing a rhizosphere metagenomic framework for optimizing crop yield under root-zone fertilization.

Future research should evaluate this conceptual framework across diverse soils and climates through multi-site and multi-season experiments. Furthermore, integrating process-based measurements, such as ^15^N isotope tracing and direct flux quantification, will be crucial to firmly link metagenomic functional potentials with in situ N transformation rates. Finally, systematically manipulating nutrient placement will help assess whether rhizosphere N–P interactions can be further leveraged to maximize plant nutrient acquisition and yield under localized fertilization strategies.

## Data Availability

The original contributions presented in this study are included in the article/Supplementary Materials. Further inquiries can be directed to the corresponding author.

## Supplementary Materials

The following supporting information can be downloaded at: https://www.mdpi.com/article/10.3390/plants15081137/s1, Figure S1: Correlations between soil mineral N (NH_4_^+^–N, NO_3_^−^–N, AN) and plant traits; Figure S2: Comparison of relative abundances at the phylum level; Table S1: List of selected N-cycling functional genes and their descriptions; Table S2: Top genera contributing to N-cycling functional modules; Table S3: Microbial co-occurrence network parameters; Table S4: Mantel test results correlating environmental factors with N-cycling functional modules; Table S5: Correlations between N-cycling functional modules and plant traits; Table S6: Estimated effects of environmental indicators on yield.
