# Supplementary material for: Root-Zone Nitrogen Fertilization Increases Oilseed Rape Yield: Reprogramming Rhizosphere N-Cycling and Strengthening Soil–Plant Coupling
Source: Plants (Basel). 2026 Apr 8;15(8):1137. doi: 10.3390/plants15081137 (PMC13119254; doi:10.3390/plants15081137)
Supplement: Supplementary file 1 [file plants-15-01137-s001.zip › plants-4141771-supplementary.pdf]

Supplementary Information for

Root-Zone Nitrogen Fertilization Increases Oilseed Rape Yield: Reprogramming Rhizosphere N-Cycling and Strengthening Soil–Plant Coupling

Liang Cheng [1], Quanjie Shen [1] and Yifan Wang [2,3,*]

1 College of Resources and Environmental Science, Yunnan Agricultural University, Kunming 650201, China

2 State Key Laboratory of Soil and Sustainable Agriculture, Institute of Soil Science, Chinese Academy of Sciences, Nanjing 211135, China

3 University of Chinese Academy of Sciences, Beijing 100049, China

* **Correspondence: wangyifan@issas.ac.cn (Y.W.)**

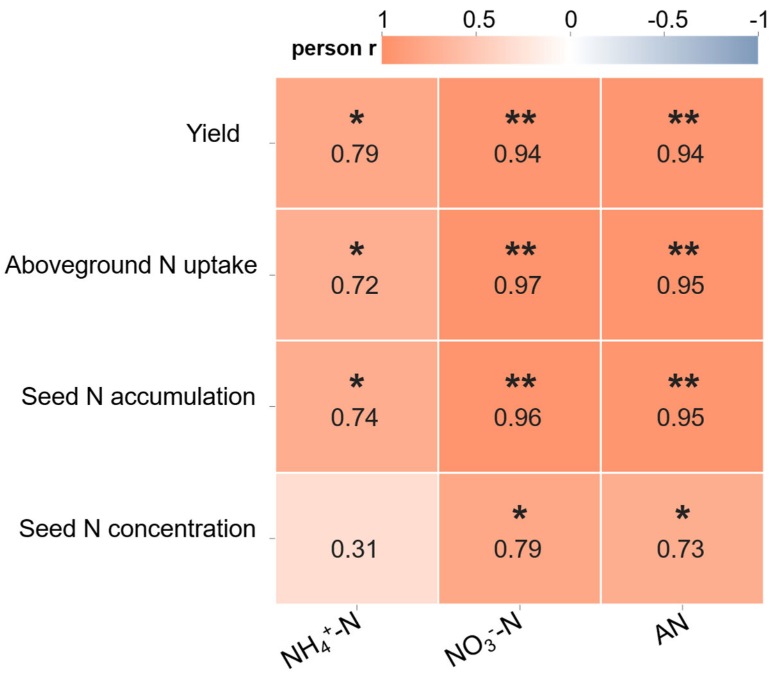

Figure S1. Correlations between soil mineral N (NH_4^+–N, NO_3^-–N, AN) and plant traits

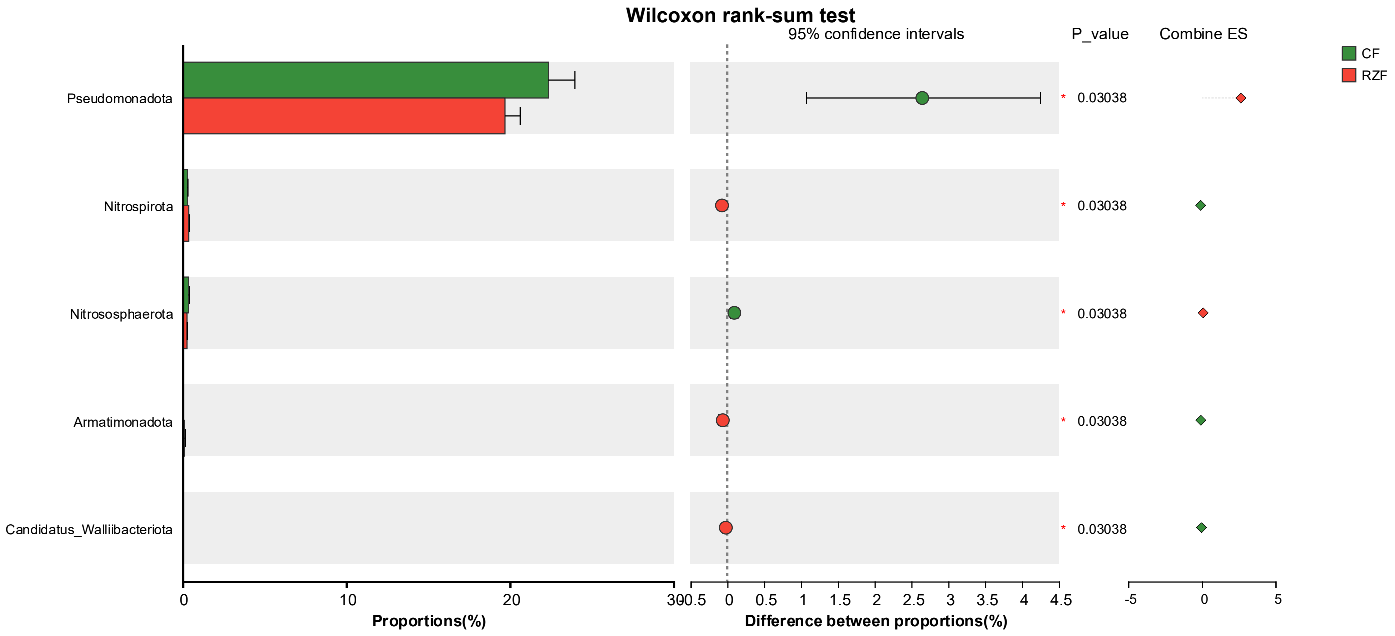

Figure S2. Comparison of relative abundances at the phylum level

Table S1. List of selected N-cycling functional genes and their descriptions

KEGG number	Gene name	Encoded protein (EC) / KO description
**Nitrogen fixation**		
K02586	nifD	nitrogenase molybdenum-iron protein alpha chain [EC:1.18.6.1]
K02588	nifH	nitrogenase iron protein NifH
K02591	nifK	nitrogenase molybdenum-iron protein beta chain [EC:1.18.6.1]
**Nitrate assimilation**		
K00261	GLUD1_2, gdhA	glutamate dehydrogenase (NAD(P)+) [EC:1.4.1.3]
K00265	gltB	glutamate synthase (NADPH) large chain [EC:1.4.1.13]
K00266	gltD	glutamate synthase (NADPH) small chain [EC:1.4.1.13]
K01915	glnA, GLUL	glutamine synthetase [EC:6.3.1.2]
**Assimilatory nitrate reduction**		
K00360	nasB	assimilatory nitrate reductase electron transfer subunit [EC:1.7.99.-]
K00366	nirA	ferredoxin-nitrite reductase [EC:1.7.7.1]
K00367	narB	ferredoxin-nitrate reductase [EC:1.7.7.2]
K00372	nasC, nasA	assimilatory nitrate reductase catalytic subunit [EC:1.7.99.-]
**Dissimilatory nitrate reduction**		
K00362	nirB	nitrite reductase (NADH) large subunit [EC:1.7.1.15]
K00363	nirD	nitrite reductase (NADH) small subunit [EC:1.7.1.15]
K02567	napA	nitrate reductase (cytochrome) [EC:1.9.6.1]
K02568	napB	nitrate reductase (cytochrome), electron transfer subunit
K02569		periplasmic nitrate reductase, electron transfer subunit
K03385	nrfA	nitrite reductase (cytochrome c-552) [EC:1.7.2.2]
K15876	nrfH	cytochrome c nitrite reductase small subunit
**Nitrification**		
K10535	hao/hzo	hydroxylamine dehydrogenase [EC:1.7.2.6]
K10944	pmoA-amoA	methane/ammonia monooxygenase subunit A [EC:1.14.18.3 1.14.99.39]
K10945	pmoB-amoB	methane/ammonia monooxygenase subunit B
K10946	pmoC-amoC	methane/ammonia monooxygenase subunit C
**Complete nitrification**		
K10535	hao	hydroxylamine dehydrogenase [EC:1.7.2.6]
**Denitrification**		
K00368	nirK	nitrite reductase (NO-forming) [EC:1.7.2.1]
K00370	narG, narZ, nxrA	nitrate reductase / nitrite oxidoreductase, alpha subunit [EC:1.7.5.1 1.7.99.-]
K00371	narH, narY, nxrB	nitrate reductase / nitrite oxidoreductase, beta subunit [EC:1.7.5.1 1.7.99.-]
K00374	narI, narV	nitrate reductase gamma subunit [EC:1.7.5.1 1.7.99.-]

K00376	nosZ	nitrous-oxide reductase [EC:1.7.2.4]
K02305	norC	nitric oxide reductase subunit C
K04561	norB	nitric oxide reductase subunit B [EC:1.7.2.5]
K15864	nirS	nitrite reductase (NO-forming) / hydroxylamine reductase [EC:1.7.2.1 1.7.99.1]
**Anammox**		
K10535	hao/hzo	hydroxylamine dehydrogenase [EC:1.7.2.6]

Table S2. Top genera contributing to N-cycling functional modules

Genera	N cycling functional	Relative abundance (%)					
		N0		CF		RZF	
Defluviicoccus	Nitrogen fixation	1.30	a	1.22	a	1.20	a
Frankia	Nitrogen fixation	0.26	a	0.21	a	0.22	a
Baekduia	Assimilatory nitrate reduction	3.54	a	2.83	b	3.00	b
Conexibacter	Assimilatory nitrate reduction	3.25	a	2.75	b	2.68	b
Nitrosospira	Nitrification	0.07	b	0.47	a	0.05	b
Thermus	Nitrification	0.11	a	0.12	a	0.11	a
unclassified_f__Nitrososphaeraceae	Nitrification	0.12	b	0.19	a	0.10	b
Capillimicrobium	Complete nitrification	2.13	a	1.78	a	1.84	a
Anaeromyxobacter	Denitrification	1.17	a	1.10	a	1.33	a
Bradyrhizobium	Nitrogen fixation, Nitrate assimilation	1.43	b	1.47	b	1.83	a
Gaiella	Nitrate assimilation, Dissimilatory nitrate reduction, Complete nitrification, Denitrification, Anammox	6.27	a	5.94	a	5.96	a
Nocardioides	Nitrate assimilation, Assimilatory nitrate reduction, Dissimilatory nitrate reduction, Complete nitrification, Denitrification, Anammox	8.07	b	11.83	a	9.15	ab
Pedococcus	Dissimilatory nitrate reduction, Anammox	2.18	a	2.24	a	2.12	a

N0, zero-N control; CF, conventional fertilization; RZF, root-zone N fertilization. Different letters indicate significant differences among treatments ($p < 0.05$).

Table S3. Microbial co-occurrence network parameters

Treatments	Nodes	Edges	Positive edges	Negative edges	Average degree	Average path length	Diameter	Density	Clustering coefficient
N0	213	272	146	126	2.55399061	2.907875895	9	0.012047	0.112612613
CF	217	286	171	115	2.6359447	3.927836414	11.89737	0.012203	0.088235294
RZF	219	321	169	152	2.931506849	2.71959883	7	0.013447	0.12244898

N0, zero-N control; CF, conventional fertilization; RZF, root-zone N fertilization.

Table S4. Mantel test results correlating environmental factors with N-cycling functional modules

Environmental factors	Nitrogen fixation		Nitrate assimilation		Assimilatory nitrate reduction		Dissimilatory nitrate reduction		Nitrification		Complete nitrification		Denitrification		Anammox	
	r	p	r	p	r	p	r	p	r	p	r	p	r	p	r	p
pH	0.47	0.01	-0.20	0.83	0.61	0.02	-0.11	0.69	0.61	0.01	0.32	0.07	0.65	0.01	0.63	0.01
OM	0.00	0.49	-0.17	0.76	-0.25	0.90	-0.30	0.92	-0.17	0.80	-0.28	0.90	-0.05	0.56	0.12	0.24
TN	0.31	0.04	0.01	0.52	0.27	0.08	-0.02	0.48	0.93	0.00	-0.13	0.72	0.23	0.10	0.10	0.31
C/N	-0.09	0.60	0.07	0.38	0.31	0.07	-0.18	0.78	0.36	0.05	-0.26	0.91	-0.08	0.63	-0.25	0.91
TP	0.26	0.25	0.26	0.31	0.07	0.29	0.22	0.25	0.56	0.00	-0.14	0.61	-0.06	0.49	0.01	0.41
AP	0.16	0.36	-0.06	0.64	0.56	0.01	0.05	0.42	0.68	0.00	0.26	0.12	0.32	0.06	0.43	0.05

OM, organic matter; TN, total N; TP, total P; AP, available P; AN, available N (NH_4^+–N + NO_3^-–N).

Table S5. Correlations between N-cycling functional modules and plant traits

N cycling functional module	Yield		Aboveground N uptake		Seed N accumulation		Seed N concentration		NH_4^+-N		NO_3^--N		AN	
	r	p	r	p	r	p	r	p	r	p	r	p	r	p
Nitrogen fixation	0.83	0.01	0.80	0.02	0.84	0.01	0.58	0.13	0.60	0.12	0.81	0.02	0.80	0.02
Nitrate assimilation	0.26	0.53	0.17	0.69	0.21	0.61	-0.13	0.75	0.51	0.20	0.27	0.52	0.31	0.46
Assimilatory nitrate reduction	0.64	0.09	0.67	0.07	0.64	0.09	0.53	0.17	0.64	0.09	0.71	0.05	0.72	0.04
Dissimilatory nitrate reduction	0.14	0.74	0.08	0.84	0.11	0.79	-0.08	0.86	0.41	0.32	0.20	0.64	0.23	0.58
Nitrification	-0.85	0.01	-0.87	0.00	-0.86	0.01	-0.72	0.05	-0.77	0.03	-0.95	0.00	-0.94	0.00
Complete nitrification	-0.30	0.47	-0.32	0.44	-0.30	0.47	-0.22	0.59	-0.06	0.89	-0.21	0.62	-0.19	0.65
Denitrification	-0.58	0.14	-0.66	0.07	-0.63	0.10	-0.75	0.03	-0.26	0.54	-0.66	0.08	-0.61	0.11
Anammox	-0.60	0.12	-0.61	0.11	-0.61	0.11	-0.51	0.20	-0.20	0.63	-0.55	0.16	-0.51	0.20

AN, available N (NH_4^+–N + NO_3^-–N).

Table S6. Estimated effects of environmental indicators on yield

Indicators	Standardized total effect	Standardized direct effect	Standardized indirect effect
N application methods	0.93	0.08	0.85
C/N	0.10	0.00	0.10
pH	0.17	0.00	0.17
R/L	0.20	0.00	0.20
AN	0.90	0.90	0.00

OM, organic matter; TN, total N; TP, total P; AP, available P; AN, available N (NH_4^+–N + NO_3^-–N); R/L, ratio of N-retention to N-loss module abundance (RPKM-based).
